# Antioxidant Peptides from the Fruit Source of the Oil Crop *Litsea cubeba* Ameliorate FFA-Induced Oxidative Stress Injury: Based on Nrf2/Keap1 Pathway and Molecular Dynamics Simulations

**DOI:** 10.3390/foods14101707

**Published:** 2025-05-12

**Authors:** Li Li, Ying Hu, Yu-Mei Wang, Xiao-Xue Wu, Si-Tong Lin, Hang Li, Ji Zhang, Guo-Rong Fan, Zong-De Wang, Bin Wang, Shang-Xing Chen

**Affiliations:** 1 East China Woody Fragrance and Flavor Engineering Technology Research Center of National Forestry and Grassland Administration, College of Forestry, Jiangxi Agricultural University, Nanchang 330045, China; lili-faye@stu.jxau.edu.cn (L.L.);; 2 Jiangxi Provincial Key Laboratory of Improved Variety Breeding and Efficient Utilization of Native Tree Species, Nanchang 330045, China; 3 Zhejiang Provincial Engineering Technology Research Center of Marine Biomedical Products, School of Food and Pharmacy, Zhejiang Ocean University, Zhoushan 316022, China

**Keywords:** *Litsea cubeba* antioxidant peptides, ROS, Keap1/Nrf2 pathway, molecular docking, molecular dynamics simulations

## Abstract

In this study, we systematically investigated the mechanisms of the antioxidation and anti-lipid accumulation effects of antioxidant peptides from *Litsea cubeba* on a free fatty acid (FFA)-induced NAFLD model of HepG2 cells. The NAFLD cell model was constructed by inducing the HepG2 hepatocellular carcinoma cell line with 0.5 mmol/L FFAs, and AQRDAGLL, QEGPFVR, and DVPPPRGPL were given to the culture to study their lipid-lowering and antioxidant activities on NAFLD cells. The lipid-lowering activities of the three antioxidant peptides were evaluated by Oil Red O staining and TG and TC content assays, and the results showed that all three peptides had strong ameliorating effects on FFA-induced lipid accumulation in NAFLD cells. The intracellular antioxidant protease (CAT, GSH, and SOD) activity levels and lipid peroxidation (MDA) content were measured and intracellular ROS levels were detected. The results showed that after intervention with the antioxidant peptides, the intracellular ROS levels in the NAFLD model cells were significantly reduced, the SOD and CAT activities were increased, the GSH content was elevated, and the MDA content was reduced, which indicated that AQRDAGLL, QEGPFVR, and DVPPPRGPL were able to inhibit the oxidative stress of the cells effectively and to achieve the effect of intervening in NAFLD. JC-1 fluorescence staining experiments showed that the mitochondrial membrane potential function of NAFLD cells was restored under the effect of the antioxidant peptides. Molecular dynamics simulations revealed that the main driving force between QEGPFVR and Keap1 protein was van der Waals forces, ΔG = −62.11 kcal/mol, which indicated that QEGPFVR was capable of spontaneously binding to Keap1 protein.

## 1. Introduction

The production and processing of oilseed crops generate large amounts of waste. Further waste processing can maximize resource utilization, minimize emissions, and promote the synergistic development of agriculture [1], animal husbandry [2], energy [3], and environmental protection. Oilseed crop meal is a byproduct of the oilseed industry and is rich in high-quality protein resources. The antioxidant peptides prepared by enzymolysis or fermentation have broad application potential due to their natural, safe, and significant activity [4]. Their hydrolysis products, small molecule peptides, can be absorbed in the intestine without degradation into amino acids [5,6]. These peptides exhibit better stability and solubility [7]. To date, antioxidant-active peptides from oilseed sources have been extensively studied. Wang et al. demonstrated the antioxidant activity of canola peptides obtained through solid-state fermentation [8]. Han et al. found that hydrolyzed alcalase-and pepsin-treated flaxseed peptides exhibited antioxidant activity [9]. Peptides derived from soybean proteins possess anti-inflammatory [10], antioxidant [11], and anticancer [12] properties. A comprehensive study on sunflower-derived bioactive peptides showed their antioxidant properties in zebrafish. Computational interactions with Keap1 indicated significant antioxidant capacity [13]. The *Litsea cubeba* fruit can be extracted to produce a high-quality oil with a strong aromatic flavor that adds texture and flavor to food [14,15]. Also, *Litsea cubeba* fruit extract has antioxidant activity [16]. With the continuous expansion of the cultivation and processing industry of *Litsea cubeba*, a large amount of waste meal is produced, which is rich in protein. Therefore, it is necessary to take *Litsea cubeba* cake meal as a raw material and develop it into a natural antioxidant, which improves the comprehensive utilization of *Litsea cubeba* resources and provides new ideas and directions for the development and utilization of agricultural and food industry wastes.

The Nrf2/Keap1 pathway is an important defense mechanism for organisms to cope with oxidative stress, and a close relationship exists between it and oxidative stress [17]. Oxidative stress is a process by which there is an imbalance between oxidative and antioxidant effects in the organism, leading to the overproduction of free radicals such as ROS and reactive nitrogen species [18] in the body or the cell, which in turn causes cell and tissue damage [19,20]. Oxidative stress causes damage to multiple systems in the organism, resulting in damage to biomolecules such as intracellular lipids, proteins, and DNA, which can lead to cellular dysfunction or disease development [19,21]. When cells are subjected to oxidative stress, the Nrf2/Keap1 pathway is activated, which can scavenge free radicals and oxidative products in the body by regulating the expression of downstream related antioxidant enzymes [22,23], including superoxide dismutase, catalase, glutathione peroxidase, etc., and alleviate cellular damage by oxidative stress, thus maintaining intracellular redox homeostasis as well as metabolic and protein homeostasis [24]. In summary, the Nrf2/Keap1 pathway is an important defense mechanism for organisms to cope with oxidative stress. Therefore, studying the Nrf2/Keap1 pathway will provide new ideas and methods for preventing and treating related diseases.

In our previous study, the protein extracted from the defatted cake meal of *Litsea cubeba* was hydrolyzed using alcalase and papain to obtain antioxidant peptides. IV2 (*Litsea cubeba* multidimensional peptide) positively influenced the viability injury in mice induced by a high-fat diet (HFD) through modulation of the Keap1/Nrf2 pathway. LC-MS/MS was used to analyze IV2, and 20 peptides were obtained. Nevertheless, the underlying interaction mechanism between IV2 and the Keap1/Nrf2 pathway remains unelucidated [25]. Based on the above evidence, we proposed the hypothesis that antioxidant peptides derived from *Litsea cubeba* cake meal could reverse the NAFLD process by inhibiting hepatic lipid peroxidation through specific activation of the Keap1-Nrf2 pathway. This study aimed to verify that antioxidant peptides from celandine promote the nuclear translocation of Nrf2 and the expression of downstream antioxidant enzymes (SOD and CAT) through competitive binding to the Keap1 Kelch structural domain; at the same time, the improvement in redox homeostasis mediated by antioxidant peptides from *Litsea cubeba* synergistically modulates the PPAR-α/CPT-1a lipid metabolism axis and reduces the accumulation of lipids in hepatocytes. In this study, we selected three peptides, AQRDAGLL, QEGPFVR, and DVPPPRGPL, with PeptideRanker scores > 0.5 for the cellular experiments, including Oil Red O and ROS staining. In recent years, computational biology methods based on molecular docking and molecular dynamics simulations have provided new ideas for elucidating the conformational relationships of peptides. We utilized molecular docking and molecular dynamics simulations to study the antioxidant peptide–Keap1 protein complex interaction mechanism and perform deeper analysis of the molecular mechanisms of the antioxidant peptides of *Litsea cubeba* involved in ameliorating oxidative stress damage.

## 2. Materials and Methods

### 2.1. Materials and Reagents

All reagents were dissolved in ultrapure water. Formic acid (FA) and acetonitrile (ACN) were purchased from Sigma-Aldrich Trading Co., Ltd. (Shanghai, China). Catalase (CAT), superoxide dismutase (SOD), malonaldehyde (MDA) [26], glutathione peroxidase (GSH-Px), total cholesterol (TC), triglyceride (TG), and ROS assay kits were purchased from Nanjing Jiancheng Bioengineering Research Institute (Nanjing, China). The Oil Red O staining kit was purchased from Beijing Solarbio Science & Technology Co., Ltd. (Beijing, China). Polyene phosphatidylcholine capsules (PPc) were purchased from Sanofi Pharmaceutical Co., Ltd. (Beijing, China). Oleic acid (OA) and palmitic acid (PA) were purchased from Aladdin Scientific Corp Co., Ltd. (Shanghai, China). The HepG2 cells and the culture medium were procured from Wuhan Punosai Life Science and Technology Co., Ltd. (Wuhan, China). The AQRDAGLL, QEGPFVR, and DVPPPRGPL peptides with 98% purity were synthesized by Shanghai Apeptide Co., Ltd. (Shanghai, China). The 96-well plates were purchased from NEST Biotechnology Co., Ltd. (Hangzhou, China).

### 2.2. Analyzing and Screening of Antioxidant Peptides

Separately, 0.1 g of defatted powder of *Litsea cubeba* cake meal and IV2 were weighed and dissolved in 2 mL of distilled water, stirred, centrifuged (9000× *g*, 10 min), and the supernatant was collected. HPLC-MS/MS (Ultimate3000—API 4000 Q TRAP, from Thermo Fisher Technology Co., Ltd., Beijing, China) was performed to identify the amino acid compositions of the cake meal and IV2. The injection volume was 5 µL and the HPLC mobile phase consisted of solutions A (water with 0.1% FA) and B (95% ACN, 0.1% FA) at a flow rate of 1 mL/min with a gradient for 20 min (0 min–20 min: 5% B–100% B; column MSLab 50AA-C18: 150 × 4.6 µm).

LC-MS/MS was used to analyze IV2, and 20 peptides with higher scores were obtained after screening by searching the target protein database for matching analysis using PEAKS Studio 10.6 [27]. Twenty peptide sequences were scored using the PeptideRanker system as a screening criterion. The higher the score, the greater the likelihood that the peptide is biologically active. The Toxin Pred system was also used to analyze the toxicity of peptides with higher bioactivity scores.

### 2.3. FFA-Induced Lipid Accumulation in HepG2 Cells

The experiments were based on the slightly modified method by Tan et al. [28]. Oleic acid and palmitic acid were configured into the FFA solution at a molar volume ratio of 2:1 for use. Logarithmically grown HepG2 cells were inoculated in 96-well plates and incubated at 37 °C in a 5% CO_2_ cell culture incubator. The cell growth was observed under the microscope. After the cells adhered to the wall and stabilized for 48 h, the blank control group was treated with 200 μL of the new unique medium. By contrast, the FFA group was treated with medium containing different FFA concentrations, which were set to 4 concentrations, respectively, 0.25, 0.5, 0.75, and 1.0 mmol/Lol/L, and the cells continued to be cultured for 24 h. The cells were then incubated for 2 h with the FFA solution at a 2:2 ratio.

### 2.4. CCK8 Experiments

The CCK8 experiments were performed according to the previous method [25]. The 96-well plates were incubated for 24 h, the medium in the wells was blotted out, 10% CCK8 solution (diluted in serum-free medium) was added to each well under the condition of light protection, and the plates were incubated in an incubator at 37 °C with 5% CO_2_ for 1 h. Afterward, each well’s optical density (OD) was measured using an enzyme labeling apparatus at a wavelength of 490 nm, and the experimental data were processed and analyzed.

### 2.5. Peptide Grouping and Administration

HepG2 cells in the logarithmic growth phase were inoculated in 96-well plates at 10,000 cells/well and placed in a 37 °C, 5% CO_2_ incubator for incubation. After the cells adhered to the wall and stabilized for 24 h, the medium was changed, and the peptide group was treated with medium containing different concentrations of peptides to make final concentrations of 50 and 200 μmol/L. After 24 h of continued incubation, the viability of the cells was measured. The cell grouping was as follows: complete medium culture for 72 h, as the control group; add FFAs (500 μmol/L) after 48 h of adherence and incubate for 24 h, as the FFA group; add PPc (50 μmol/L) after 24 h of wall affixation and incubate for 24 h, then add FFAs (500 μmol/L) for 24 h, as the PPc group; after 24 h of wall affixation, add peptides (50 and 200 μmol/L) and incubate for 24 h, and then add FFAs (500 μmol/L) and incubate for 24 h, as the peptide group. The peptide group was incubated for 24 h with FFAs (500 μmol/L).

### 2.6. Oil Red O Staining

HepG2 cells were inoculated in 12-well plates at a density of 300,000/well, modeled by treatment with different concentrations of FFA solutions, and divided into five groups: the control group (blank control group) and the 0.25, 0.5, 0.75, and 1.0 mmol/Lol/L FFA-treated groups. After 24 h, the cell culture medium was aspirated, and the cells were rinsed three times using sterilized PBS. An appropriate amount of 4% paraformaldehyde was added to each well for fixation, which lasted for 30 min at room temperature. The residue was washed by pouring out the paraformaldehyde, washing the residue with PBS, adding 60% Oil Red O staining solution, and left to stand at room temperature for 60 min. Subsequently, the isopropanol was differentiated for 10 s, followed by PBS rinsing 3 times. The accumulation of intracellular lipid droplets was observed using a microscope, and photographs were recorded.

### 2.7. TG and TC Content

Cell lysis was performed according to the kit instructions, and TG and TC levels in the lysate were determined.

### 2.8. Antioxidant Kit Content Assay

SOD, GSH, CAT, and MDA activities were measured according to the instructions of the kits.

### 2.9. ROS Staining

ROS staining experiments were performed according to the kit’s instructions. Cells were inoculated in 12-well plates, and 300,000 cells were placed in each well. After the cells adhered to the wall and were stably incubated for 24 h, the cell culture medium was poured off, and DCFH-DA was diluted to a final concentration of 10 μmol/L at a ratio of 1:1000 and added to the wells. The healthy plates were incubated in a cell culture incubator under light protection for 60 min. Subsequently, the cells were rinsed with PBS thrice to remove the DCFH-DA staining solution that had not entered the cells. The DCFH-DA fluorescence intensity was detected using the 488 nm excitation wavelength and 525 nm emission wavelength of the zymograph. Images were captured using a fluorescence microscope to observe the fluorescence intensity of DCFH-DA, and photographs were taken for documentation.

### 2.10. JC-1 Staining

JC-1 staining was performed according to the kit’s instructions. Aspirate the cell culture medium, wash the cells twice with PBS, add 1 mL of cell culture medium and 1 mL of JC-1 staining solution, mix well, and incubate at 37 °C with 5% CO_2_ for 20 min. After incubation, aspirate the supernatant, wash the cells twice with JC-1 staining buffer (1×), add 2 mL of JC-1 staining buffer (1×) each time, and gently shake the plate before aspirating the supernatant. Add 2 mL of cell culture solution, observe and take pictures under a fluorescence microscope, and observe the fluorescence intensity changes using ImageJ (1.8.0).

### 2.11. RT-qPCR Analysis

RNA was extracted from HepG2 cells using Trizol reagent (Beijing, China) following the instructions provided with the kit. RT-qPCR was conducted using a PCR instrument (Eastwin Life Sciences Inc., Beijing, China, ETC811) and a Real-Time PCR System (Bio-RadLaboratories Co., Ltd., Shanghai, China, CFX Connect). The primer sequences of Nrf2, HO-1, NQO1, MCJ, PPAR-α, and CPT-1a are listed in Table 1.

### 2.12. Molecular Docking and Molecular Dynamics Simulation

The coordinates of the docking pockets were determined based on the position of the owning ligand in the crystallographic structure of Keap1, based on a 20 Å BOX with center coordinates of (−2.31, 6.16, 1.65) for lattice point calculations. Each ligand was subjected to semiflexible docking in standard precision (SP-peptide) mode to generate 1000 docking poses, and the one conformer that met the expectations was selected for molecular dynamics simulations based on mold conformational scoring and structural clustering [29].

The structural stability and dynamic trajectory behavior of the complex formed by the peptide QEGPFVR with Keap1 protein were evaluated by performing 100 ns classical molecular dynamics simulations using the GPU-accelerated Desmond-v7.2 (D. E. Shaw Research, New York, NY, USA) high-performance software package. AutoMD scripts were automatically submitted to the e-Science Center of Collaborative Innovation Center of Advanced Microstructures (Nanjing, China) to the GPU acceleration queue. Using the OPLS_2005 force field, an explicit solvent simulation filled with a single point charge (SPC) water model and 0.15 mol/L NaCl, with additional Na^+^ or Cl^−^ ions to help neutralize the overall net charge of the system, was performed at a distance of 10 Å from the cubic periodic boundary condition. The solvated simulated system was subjected to a 200 ps Brownian Dynamics NVT system and multi-step Langevin thermostat and Langevin barostat simulations to remove system strain. This was followed by a 298 K NPT simulation with 1000 frames sampled for trajectory analysis. The analysis parameters included RMSD (root mean square deviation), RMSF (root mean square fluctuation), SASA (solvent accessible surface area, binding_sasa.py), and RoG (radius of gyration, calc_radgyr.py) [29].

### 2.13. Statistical Analysis

All data are expressed as the mean ± SD (n = 3) and were analyzed by an ANOVA test using SPSS 27.0.1. Duncan’s multiple range test was used to analyze significant differences between the means of parameters (*p* < 0.05, *p* < 0.01, or *p* < 0.001).

## 3. Results

### 3.1. Identification of Antioxidant Peptides

**Figure 1:**
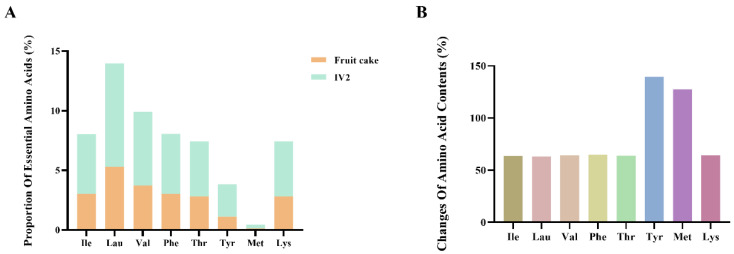
Comparison of essential amino acid contents of *Litsea cubeba* cake meal and IV2. (**A**) Specific values of essential amino acid contents. (**B**) Analysis of increased content of essential amino acids in IV2.

As shown in Figure 1A, the essential amino acid content of purified *Litsea cubeba* antioxidant peptide IV2 was significantly higher than that of *Litsea cubeba* cake meal. The contents of branched-chain amino acids Ile, Leu, and Val increased by 63.77%, 63.20%, and 64.27%, respectively (Figure 1B). The increased content of branched-chain amino acids enhances the nutritional value of antioxidant peptides and regulates intracellular redox signaling pathways. Lau plays a vital signaling role in this process, which can promote the activation of Nrf2 and improve the resistance of cells to oxidative damage [30]. Meanwhile, the content of Met was elevated by 127.56%, and methionine is involved in the synthesis of glutathione, an important intracellular antioxidant [31]. Thus, the antioxidant peptide IV2 obtained after isolation and purification was effectively enriched in essential amino acids, which significantly enhanced its nutritional value and antioxidant activity.

Based on the results of a previous study, we scored 20 peptides with high confidence scores using the PeptideRanker system as a screening criterion for peptide sequences. The study demonstrated the possible bioactive potential of the peptides with a PeptideRanker score greater than 0.5 [32]. From the 20 peptides identified, three peptides, AQRDAGLL, QEGPFVR, and DVPPPRGPL, with scores greater than 0.5 were screened. The hydrophobicity ratios of these three peptides were evaluated using the PEPTIDE 2.0 system. Their toxicity analysis was performed using the Toxin Pred system, and the results are shown in Table 2. In low-molecular-weight antioxidant peptides, hydrophobic amino acids such as leucine, isoleucine, and valine can make the peptide chain more easily exposed to the solution, enabling efficient contact and scavenging of free radicals. At the same time, these amino acids facilitate the interaction of peptides with lipid molecules, which can better inhibit lipid peroxidation and reduce the generation of oxidation products by binding lipid peroxides or scavenging related free radicals to protect cells and tissues from oxidative damage. Therefore, we hypothesized that AQRDAGLL, QEGPFVR, and DVPPPRGPL might have better biological activity, so we chose these three peptides for subsequent cellular experiments.

### 3.2. Effect of Concentration of FFAs and Peptides on the Activity of HepG2 Cells

**Figure 2:**
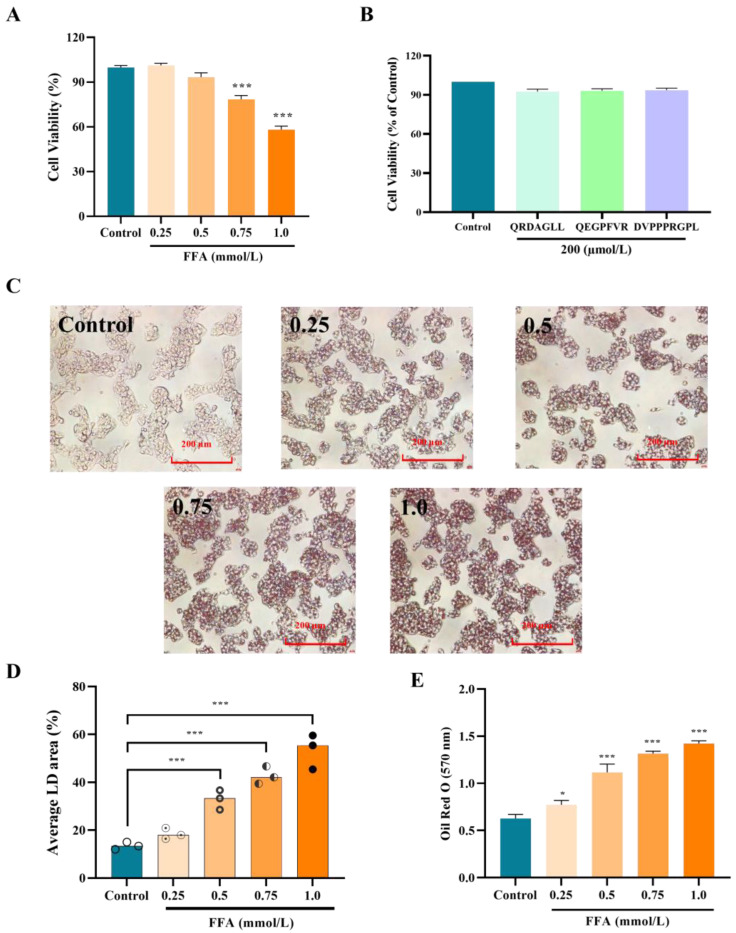
Effect of concentration of FFAs and peptides on the activity of HepG2 cells. (**A**) Effect of FFA concentration on the activity in HepG2 cells; (**B**) effect of antioxidant peptides on the activity in HepG2 cells; (**C**) effect of FFAs on intracellular lipid accumulation in HepG2 cells; (**D**) change in lipid droplet area; and (**E**) change in absorbance. * *p* < 0.05, *** *p* < 0.001 vs. Control, n = 3.

After treatment of HepG2 cells with the FFA and peptide solutions, cell viability was determined using the CCK8 method, and the results are shown in Figure 2A. As the concentration of FFAs increased, the activity of HepG2 cells gradually decreased. The cell viability of HepG2 cells treated with 0.5 mmol/L FFAs was 93.34% ± 2.92%, and the difference was insignificant compared with that of the blank group (*p* > 0.05). After 0.75 mmol/L and 1.0 mmol/L FFA treatment, the cell viability was significantly reduced (*p* < 0.001) to 78.48 ± 2.52% and 58.17 ± 2.36%, respectively. Therefore, there was no significant toxic effect on HepG2 cells when the concentration of FFAs was lower than 0.5 mmol/L. Similarly, AQRDAGLL, QEGPFVR, and DVPPPRGPL solutions at 200 µmol/L had no toxic effect on HepG2 cells (Figure 2B).

HepG2 cells were treated with the addition of different concentrations of FFAs. The Oil Red O staining results are shown in Figure 2C. No apparent red lipid droplets were observed in the field of view of HepG2 cells in the control group. By contrast, an increase in the area of red lipid droplets in the field of view was observed after the addition of different concentrations of FFAs. The difference was significant when compared with that in the control group. The intracellular red lipid droplet area gradually increased with the increase in FFA concentration (Figure 2D), showing a dose dependence (*p* < 0.001). Meanwhile, the measured absorbance was consistent with the trend of the lipid droplet area (Figure 2E). A large number of red lipid droplets could be observed in 0.5 mmol/L FFA-treated cells under the field of view, indicating that this FFA concentration successfully induced intracellular lipid deposition.

### 3.3. Antioxidant Peptides Improve Lipid Accumulation in HepG2 Cells

**Figure 3:**
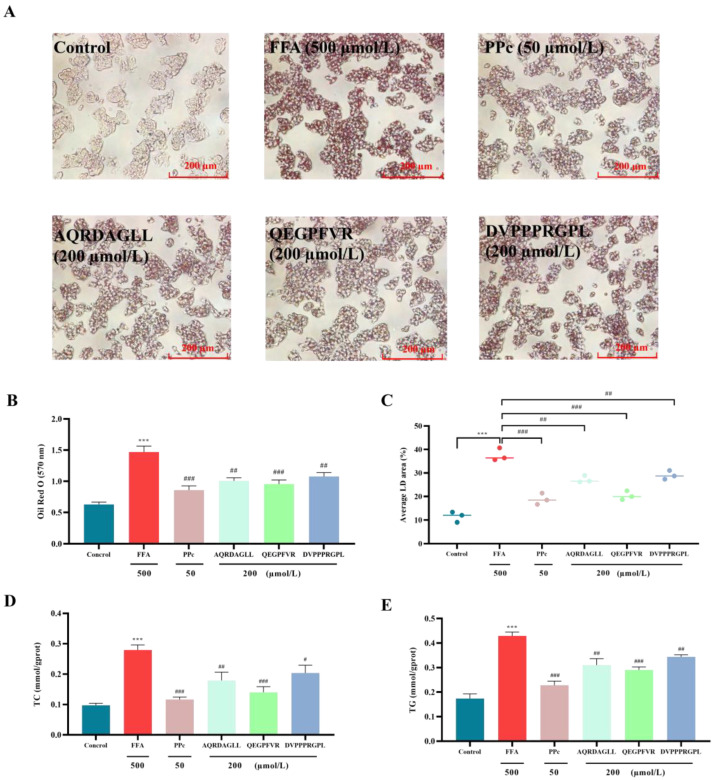
(**A**) Effect of antioxidant peptides on intracellular lipid; (**B**) change in absorbance; (**C**) change in lipid droplet area; and (**D**,**E**) TC and TG contents. *** *p* < 0.001 vs. Control; ^#^ *p* < 0.05, ^##^ *p* < 0.01, ^###^ *p* < 0.001 vs. FFA, n = 3.

To assess the effects of AQRDAGLL, QEGPFVR, and DVPPPRGPL on FFA-induced intracellular lipid accumulation in HepG2 cells, we performed Oil Red O staining (Figure 3A). Following treatment, the stained lipid droplets were dissolved in isopropanol, and the absorbance was measured at 570 nm. Compared to the FFA-treated model group, cells exposed to antioxidant peptides showed reduced lipid droplet formation, as indicated by decreased Oil Red O staining intensity. This trend was further supported by the quantitative absorbance measurements (Figure 3B,C), which correlated with the observed decrease in lipid droplet accumulation. Notably, cells treated with 0.5 mmol/L FFAs displayed prominent red-stained lipid droplets, confirming significant intracellular lipid deposition. Compared with the FFA group, after intervention with the antioxidant peptides AQRDAGLL, QEGPFVR, and DVPPPRGPL, the absorbance of Oil Red O decreased to 1.01 ± 0.05 (*p* < 0.01), 0.96 ± 0.06 (*p* < 0.001), and 1.08 ± 0.06 (*p* < 0.01), respectively. Meanwhile, the lipid droplet area decreased to 27.23% ± 1.50 (*p* < 0.01), 20.36% ± 1.91 (*p* < 0.001), and 29.03% ± 1.87 (*p* < 0.01), respectively. Together, these results demonstrated that both high and low doses (50 µmol/L) of antioxidant peptides effectively attenuated FFA-induced lipid accumulation in HepG2 cells in a dose-dependent manner.

Intracellular cholesterol and triglyceride levels were measured using TC and TG assay kits (Figure 3D,E). Treatment with 0.5 mmol/L FFAs significantly increased intracellular cholesterol and triglyceride accumulation in HepG2 cells compared to the control group (*p* < 0.001). However, co-treatment with 200 µmol/L of the antioxidant peptides AQRDAGLL, QEGPFVR, and DVPPPRGPL markedly attenuated these effects, leading to significant reductions in both cholesterol and triglyceride levels relative to the FFA-treated group. The intracellular TC content decreased to 0.18 ± 0.03 mmol/gprot (*p* < 0.01), 0.14 ± 0.02 mmol/gprot (*p* < 0.001), and 0.20 ± 0.02 mmol/gprot (*p* < 0.05) after intervention with the antioxidant peptides AQRDAGLL, QEGPFVR, and DVPPPRGPL, respectively, as compared with the FFA group. Meanwhile, the TG content decreased to 0.31 ± 0.03 mmol/gprot (*p* < 0.01), 0.29 ± 0.01 mmol/gprot (*p* < 0.001), and 0.34 ± 0.01 mmol/gprot (*p* < 0.01), respectively. These findings indicated that both low-dose and high-dose (200 µmol/L) antioxidant peptides effectively mitigated FFA-induced lipid accumulation in HepG2 cells.

### 3.4. Antioxidant Peptides Ameliorate Oxidative Damage Induced by Lipid Accumulation

**Figure 4:**
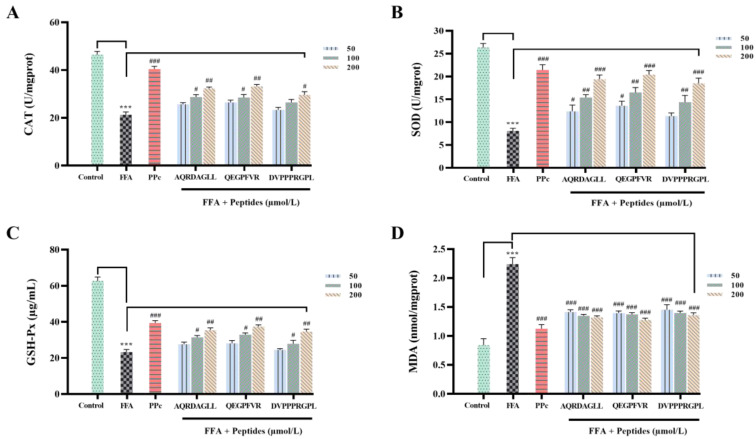
Effect of antioxidant peptides on the levels of (**A**) CAT; (**B**) SOD; (**C**) GSH-Px; and (**D**) MDA in HepG2 cells. *** *p* < 0.001 vs. Control; ^#^ *p* < 0.05, ^##^ *p* < 0.01, ^###^ *p* < 0.001 vs. FFA, n = 3.

The antioxidant effects of AQRDAGLL, QEGPFVR, and DVPPPRGPL on HepG2 cells were assessed after the measurement of SOD, CAT, GSH-Px, and MDA in each group of cells, and the results are shown in Figure 4A–D. We observed that the FFA-treated HepG2 cells had significantly lower (*p* < 0.001) CAT, SOD, and GSH-Px activities and higher (*p* < 0.001) MDA values compared to the control group. Whereas, after AQRDAGLL, QEGPFVR, and DVPPPRGPL intervention, the activities of CAT, SOD, and GSH-Px of HepG2 cells in the antioxidant peptide-treated groups were significantly increased compared to the FFA group. Among them, the CAT activity values in the high-dose administration group were increased by 51.73% (*p* < 0.01), 55.41% (*p* < 0.01), and 39.08% (*p* < 0.05), respectively; the SOD activity values were increased by 1.4-fold (*p* < 0.001), 1.52-fold (*p* < 0.001), and 1.29-fold (*p* < 0.001), respectively; and the activity values of GSH-Px were increased by 52.56% (*p* < 0.01), 61.27% (*p* < 0.01), and 49.07% (*p* < 0.01), respectively; whereas the content of MDA was significantly decreased by 40.96% (*p* < 0.001), 42.91% (*p* < 0.001), and 39.27% (*p* < 0.001), respectively. The above results indicated that AQRDAGLL, QEGPFVR, and DVPPPRGPL effectively enhanced the antioxidant capacity of cells and alleviated the cellular oxidative damage caused by peroxidation.

### 3.5. Effects of Antioxidant Peptides on ROS Levels in HepG2 Cells

**Figure 5:**
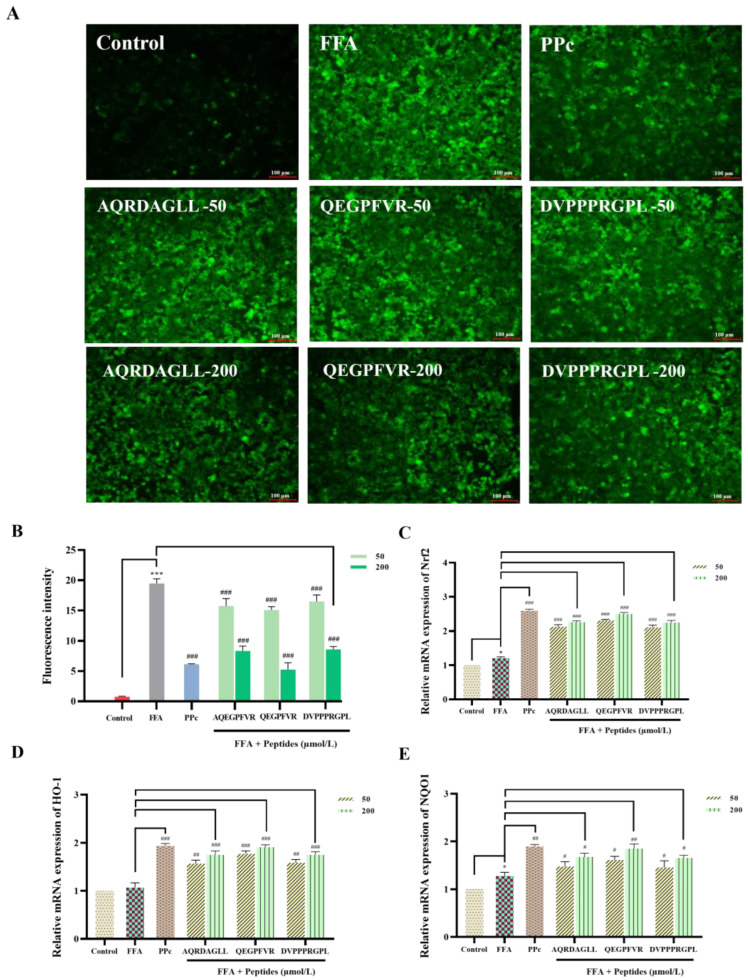
(**A**) Effect on intracellular ROS (100 µm); (**B**) change in absorbance; and (**C**–**E**) effect on the mRNA expression levels of Nrf2, HO-1, and NQO1 in cells under oxidative stress. * *p* < 0.05, *** *p* < 0.001 vs. Control; ^#^ *p* < 0.05, ^##^ *p* < 0.01, ^###^ *p* < 0.001 vs. FFA, n = 3.

The effects of AQRDAGLL, QEGPFVR, and DVPPPRGPL on FFA-induced ROS levels in HepG2 cells are shown in Figure 5A,B. After DCFH-DA staining, the fluorescence intensity of the FFA group increased significantly compared to the control group, indicating a significant increase in the intracellular ROS content of HepG2 cells in this group. By contrast, the ROS levels of AQRDAGLL, QEGPFVR, and DVPPPRGPL groups decreased in fluorescence intensity with the increase in antioxidant peptide concentration, indicating a significant decrease in intracellular ROS (*p* < 0.001). The above experimental results indicated that the antioxidant peptides AQRDAGLL, QEGPFVR, and DVPPPRGPL were effective in reducing the intracellular ROS content in FFA-induced HepG2 cells at different concentrations, regulating the balance of redox reactions and decreasing oxidative damage.

The RT-qPCR results are shown in Figure 5C–E. The mRNA expression levels of Nrf2, HO-1, and NQO1 were increased in the FFA group compared to the control group. By contrast, Nrf2, HO-1, and NQO1 expression significantly increased relative to the FFA group after intervention with AQRDAGLL, QEGPFVR, and DVPPPRGPL (*p* < 0.01). These results suggested that these three antioxidant peptides may activate the transcription of these genes by regulating the Nrf2/Keap1 pathway and activating the ARE sequence on the promoters of Nrf2 and downstream genes such as HO-1 and NQO1. Especially after high-dose intervention with QEGPFVR, the expression of HO-1 and NQO increased significantly (*p* < 0.05, *p* < 0.01). These results demonstrated that these three antioxidant peptides, especially QEGPFVR, could exert antioxidant effects by promoting Nrf2 entry into the nucleus, upregulating the expression of downstream oxidoreductases HO-1 and NQO1 and maintaining the cellular redox state.

### 3.6. Protective Effects of Antioxidant Peptides on Mitochondria

**Figure 6:**
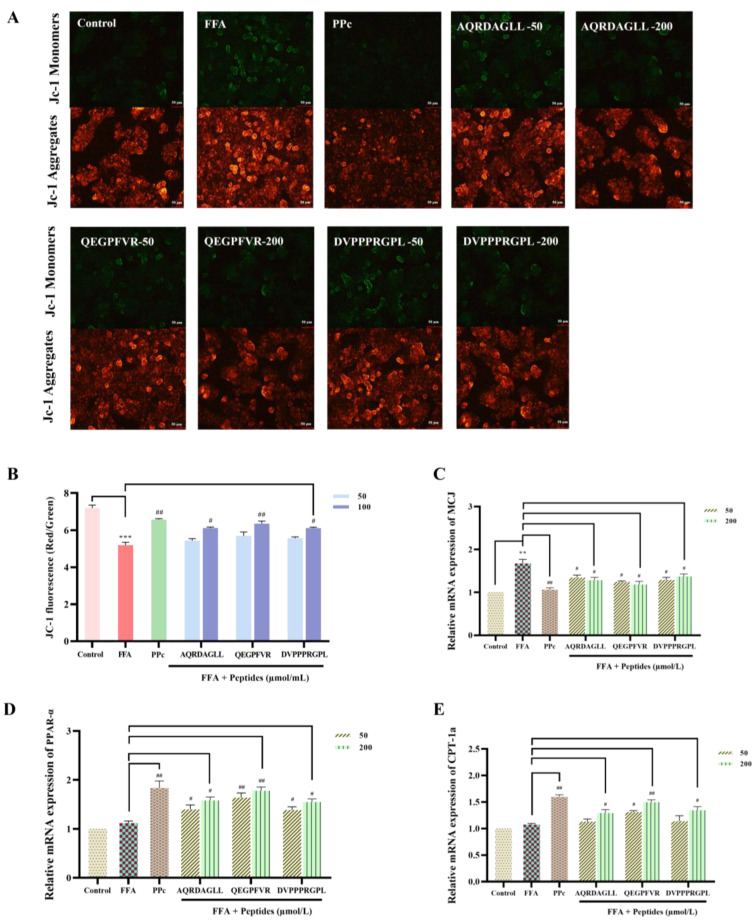
(**A**) Detection of mitochondrial membrane potential by JC-1 staining (50 µm); (**B**) fluorescence intensity change; and (**C**–**E**) effect on the mRNA expression levels of MCJ, PPAR-α, and CPT-1a in HepG2 cells. ** *p* < 0.01, *** *p* < 0.001 vs. Control; ^#^ *p* < 0.05, ^##^ *p* < 0.01. vs. FFA, n = 3.

A large amount of FFAs in HepG2 cells exceeds the standard oxidative capacity of mitochondria, causing dysfunction of the mitochondrial respiratory chain, which leads to a decrease in the mitochondrial membrane potential [33]. JC-1, a cationic lipid fluorescent dye, detects potential changes in the mitochondrial transmembrane site. Standard mitochondrial membrane potential is polarized, and JC-1 depends on the membrane potential’s polarity and aggregates to form multimers that emit red fluorescence [34]. When the mitochondrial membrane potential is disrupted and depolarization occurs, JC-1 is released from mitochondria and exists as monomers within the cell, emitting green fluorescence. Therefore, changes in JC-1 staining fluorescence can be observed to detect changes in the mitochondrial membrane potential [35]. As shown in Figure 6A,B, the FFA-induced mitochondrial membrane potential decreased, and the green fluorescence intensity increased in HepG2 cells. However, after intervention with AQRDAGLL, QEGPFVR, and DVPPPRGPL, the green fluorescence intensity in HepG2 cells was significantly reduced in a dose-dependent manner, indicating that the antioxidant peptides restored the mitochondrial membrane potential in FFA-induced HepG2 cells to normal levels.

Dysfunctional lipid metabolism leads to mitochondrial dysfunction [36]. MCJ proteins are endogenous negative regulators of the mitochondrial respiratory chain, localized in the inner mitochondrial membrane, preferentially interacting with complex I of the electron transport chain, hindering supercomplex formation, and inhibiting complex I activity, which in turn affects the mitochondrial membrane potential and ATP production [37]. PPAR-α plays a key role in fatty acid metabolism and alleviates mitochondrial dysfunction. When mitochondrial dysfunction occurs, fatty acid oxidation is often impaired. PPAR-α can activate fatty acid transporter protein (FATP) and fatty acid binding protein (FABP) to promote fatty acid entry into the cell and transport to mitochondria [38]. Meanwhile, PPAR-α is able to upregulate CPT-1a to ensure the smooth entry of fatty acids into mitochondria for β-oxidation. The RT-qPCR results showed (Figure 6C–E) that the mRNA expression level of MCJ was elevated in FFA-induced HepG2 cells. By contrast, the mRNA expression level of MCJ was significantly decreased after intervention with AQRDAGLL, QEGPFVR, and DVPPPRGPL, decreasing by 23.44% (*p* < 0.05), 29.29% (*p* < 0.05), and 18.17% (*p* < 0.05), respectively (200 µmol/L). In addition, the mRNA expression levels of PPAR-α and CPT-1a were significantly increased in cells after intervention with the antioxidant peptides, and the PPAR-α expression was 41.31% (*p* < 0.05), 59.04% (*p* < 0.01), and 38.35% (*p* < 0.05) higher, respectively (200 µmol/L). Meanwhile, the CPT-1a expression was elevated by 20.17% (*p* < 0.05), 53.28% (*p* < 0.01), and 25.15% (*p* < 0.05), respectively (200 µmol/L). We hypothesized that improving mitochondrial function by treatment with the antioxidant peptides may affect the expression of MCJ, PPAR-α, and CPT-1a. After the antioxidant peptides downregulated MCJ expression by scavenging ROS and attenuating oxidative stress, mitochondrial respiratory chain complex I activity was restored, and mitochondrial function was improved. This synergized with the enhanced effect of fatty acid oxidation brought about by the upregulation of PPAR-α and CPT-1a, further optimizing mitochondrial energy metabolism. The antioxidant peptides restored the normal function of the mitochondrial respiratory chain and increased the mitochondrial membrane potential and ATP production. Restoration of the mitochondrial membrane potential, as revealed by JC-1 fluorescent staining, signified the reestablishment of mitochondrial energy metabolism and redox homeostasis, a process that can directly enhance CPT1-mediated fatty acid transport and β-oxidase activity. When the antioxidant peptides reduced the level of oxidative stress, the intracellular environment became more favorable for activating the PPAR-α/CPT-1a signaling pathway. Moreover, the upregulation of PPAR-α and CPT-1a could enhance the oxidative metabolism of fatty acids and reduce lipid accumulation.

### 3.7. Molecular Docking of QEGPFVR with Keap1

#### 3.7.1. Molecular Docking Results

**Figure 7:**
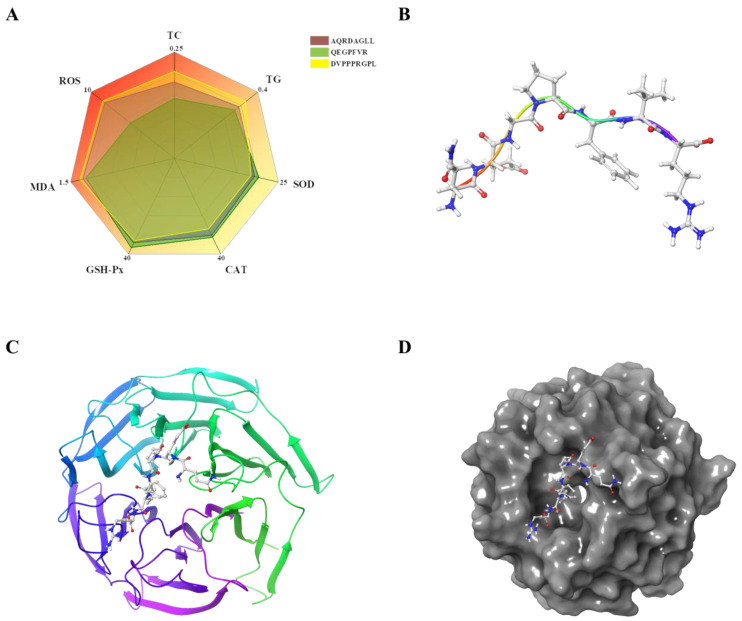
Molecular docking modeling of QEGPFVR with Keap1 (kelch). (**A**) Six-axis spider web graph of the properties of the antioxidant peptide; (**B**) structure of peptide QEGPFVR; (**C**) color band diagram of Keap1 protein docking complex with peptide QEGPFVR; and (**D**) waveform.

Based on the effects of the three antioxidant peptides on lipid accumulation and oxidative stress injury in HepG2 cells, we observed that QEGPFVR possessed better lipid-lowering and antioxidant activities. Its ability to regulate the expression level of the Keap1/Nrf2 pathway was relatively significant (Figure 7A). To verify the antioxidant effect of QEGPFVR and investigate its inhibitory mechanism deeply, we used a molecular docking method to conduct the study and confirm the role of QEGPFVR in terms of the binding affinity and mode of the QEGPFVR–Keap1 conjugate. The structure of the antioxidant peptide QEGPFVR is shown in Figure 7B. Figure 7C,D shows the Keap1 protein docking complex with the peptide QEGPFVR. Compared to the Keap1 protein’s ligand, QEGPFVR is also located in the center of the active pocket of Keap1 protein, and upon binding, the position of the QEGPFVR structure changes, indicating that the QEGPFVR–Keap1 protein complex restructures to a more stable state.

#### 3.7.2. Combined Model Analysis

**Figure 8:**
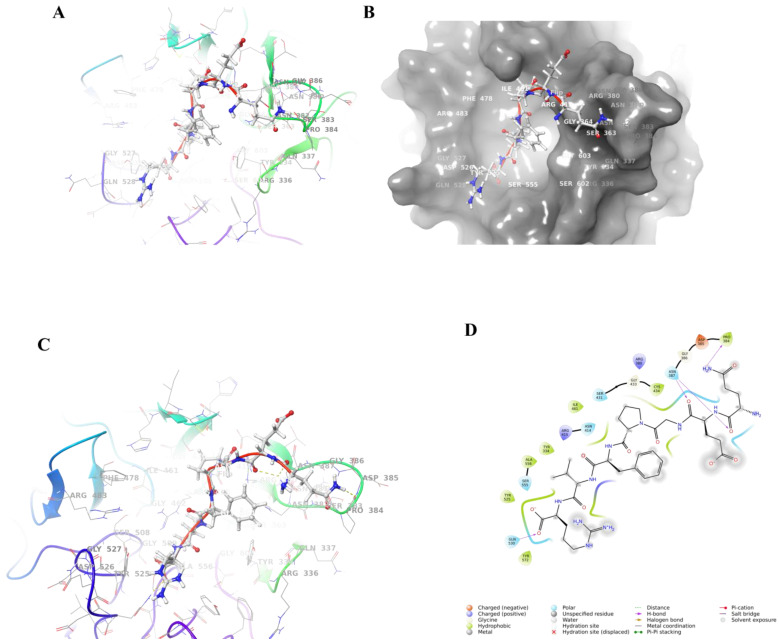
Localized pocket details of the Keap1 protein docking complex with peptide QEGPFVR. (**A**) Waveform; (**B**) QEGPFVR–Keap1 protein complex localized pocket details color band diagram; (**C**) waveform; and (**D**) QEGPFVR–Keap1 protein complex 3D interaction force demonstration. Yellow dashed lines are hydrogen bonds, and blue dashed lines are pi–pi conjugation.

The Keap1-Kelch structural domain is a six-bladed β-propeller, with each blade containing a four-stranded antiparallel β-sheet connected by rings of different lengths [39]. The shorter loops connecting the β-strands A-B and C-D are exposed on the bottom surface of the β-propeller. By contrast, the longer loops connecting the β-strands D-A and B-C extend toward the top surface, sculpting an active pocket that Nrf2 can recognize [40]. The binding pocket of the Kelch structural domain is divided into six sub-pockets (P1-P6) [41]. Subpockets P1 (Arg415, Ile461, Gly462, Phe478, Arg483, Ser508) and P2 (Ser363, Arg380, Asn382, Asn414) are strongly polar, while P4 (Tyr525, Gln530) and P5 (Tyr334, Tyr572, Phe577) are hydrophobic. P3 (Gly509, Ser555, Ala556, Gly571, Ser602, Gly603) is located in the center of the channel formed by small polar residues. P6 (Asp389, Ser431, His432, Gly433, Cys434, Ile435, His436) is in the center of the channel formed by Keap1 and these residue interactions were not observed in the ETGE motif [42,43]. Based on the localized pocket details of the Keap1 protein–peptide QEGPFVR docking complex (Figure 8A–D), it was possible to observe that QEGPFVR is deeply embedded in the active pocket of Keap1 protein. In particular, QEGPFVR binds to Tyr 572, Tyr 525, Ala 556, Tyr 334, Ile 461, Cys 434, and Pro 384 residues in the active pocket of Keap1 protein with water-transporting forces; to Gln 530, Ser 555, Asn 414, Ser 431, and Asn 387 residues in the active pocket through hydrogen bonding; while binding to Arg 415, Asp 385, and Arg 380 residues in the active pocket through electrostatic interactions. The results indicated that the QEGPFVR–Keap1 protein complex possessed more hydrophobic interactions, hydrogen-bonding interactions, and electrostatic interactions, which could effectively reduce the effect of unfavorable forces on stability. These results suggested that QEGPFVR–Keap1 can form a more stable complex.

### 3.8. Molecular Dynamics Simulation of Keap1 Protein with QEGPFVR

#### Stability Analysis of QEGPFVR–Keap1 Protein Complexes

**Figure 9:**
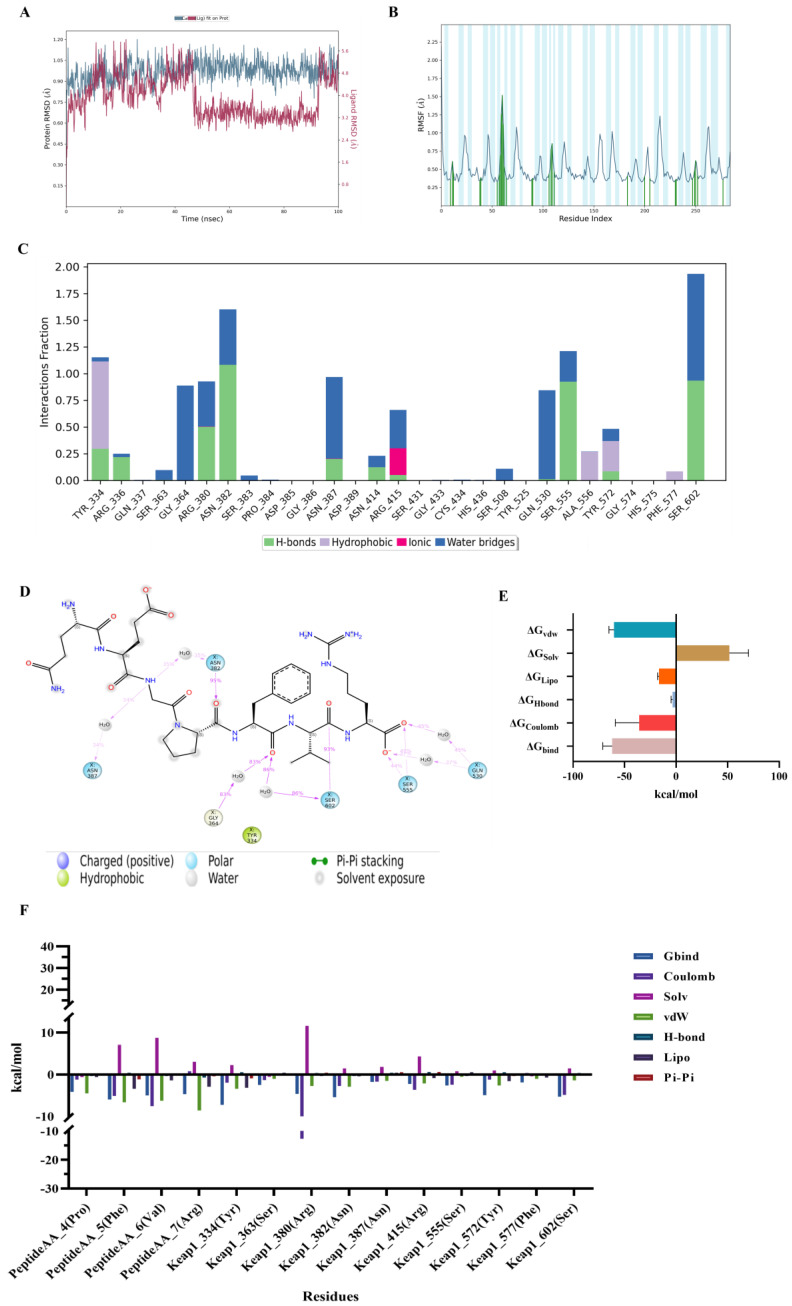
Molecular dynamics simulations of QEGPFVR–Keap1 protein complex. (**A**) RMSD (blue lines indicate Keap1 protein, red lines indicate QEGPFVR–Keap1 protein complex); (**B**) RMSF (the blue background highlights the beta chain, which persists throughout the simulation for more than 70% of the time); (**C**) Interaction of QEGPFVR with Keap1 protein (green for hydrogen bonding; purple for water transport; pink for ionic bonding; blue for water bridges); (**D**) A schematic of detailed QEGPFVR interactions with Keap1 protein residues; (**E**) Calculation of the binding free energy of QEGPFVR to Keap1 protein; (**F**) Contribution of individual amino acids to the binding energy of QEGPFVR to Keap1 protein.

The root mean square deviation (RMSD) indicates the sum of all atomic deviations of the conformation from the target conformation at a given moment in time, and the smaller its fluctuation, the more stable the system will be [44]. Figure 9A shows the RMSD evolution of Keap1 protein (left Y-axis). The variation in Keap1 protein is concentrated between 0.1 and 0.4 Å, indicating that the Keap1 protein system is stable. The ligand RMSD (right Y-axis) indicates the stability of QEGPFVR relative to Keap1 protein and its binding pocket. It can be observed that the RMSD values of the QEGPFVR–Keap1 protein complex fluctuated around 3–6 Å between 15–45 ns and 90–100 ns and around 2.8–4 Å between 45 and 90 ns, which suggested that the relative position of the QEGPFVR–Keap1 protein complex varied at 15 ns, 45 ns, and 90 ns and stabilized after 90 ns, with its RMSD value fluctuating around 4.8 nm. This was due to the nature of QEGPFVR itself and its interaction with amino acid residues in the active pocket center of Keap1 protein. The RMSD value of the QEGPFVR–Keap1 protein complex did not undergo any significant changes relative to the initial structure, suggesting that it could remain stable during the simulation process. The root mean square fluctuation (RMSF) can indicate the flexibility size of amino acid residues in proteins, and higher values indicate better flexibility of amino acid residues [45]. As shown in Figure 9B, the peaks indicate the Kelch region with the most prominent fluctuation during the simulation, and the Keap1 protein residues interacting with QEGPFVR are marked with green vertical bars. The RMSF values of the QEGPFVR–Keap1 protein complexes ranged from 5–10, 40–45, 50–60, 80–90, 100–120, 200–210, 230–235, and 245–255, indicating that amino acid residues in these active pockets actively participated in the binding process of QEGPFVR to Keap1 protein and formed the QEGPFVR–Keap1 protein complex by restructuring its conformation.

We monitored the interactions of QEGPFVR with Keap1 protein throughout the simulation and categorized and summarized the results by type, as shown in Figure 9C. Residues Tyr 334 and Tyr 572 formed a hydrophobic bond, hydrogen bond, and water bridge interactions with QEGPFVR; residues Arg 336, Arg 380, Asn 382, Asn 387, Asn 414, Gln 530, Ser 555, and Ser 602 interacted with QEGPFVR through hydrogen bonds and water bridges; and Arg 415 interacted with QEGPFVR through an ionic bond, hydrogen bonding, and water bridge interactions; Ser 363, Gly 364, Ser 383, and Ser 508 interacted with QEGPFVR through water bridges; and Ala 556 and Phe 577 were connected to QEGPFVR with water transport. Figure 9D shows a detailed schematic of the interactions of QEGPFVR with Keap1 protein residues. For stacked bars standardized throughout the trajectory, 0.7 indicates that a specific interaction was maintained for 70% of the simulation time. Values above 1.0 may be because particular protein residues formed multiple contacts of the same isoform with the ligand. It could, therefore, be hypothesized that residues Tyr 334, Arg 336, Arg 380, Asn 382, Asn 387, Asn 414, Arg 415, Gln 530, Ser 555, Tyr 572, and Ser 602 made more than one specific contact with QEGPFVR.

To obtain information about the energy of the QEGPFVR–Keap1 protein complex, we calculated the binding energy of the QEGPFVR–Keap1 protein complex. The results of the energy calculation for the QEGPFVR–Keap1 protein complex after interaction are shown in Figure 9E, with a van der Waals energy (ΔG_vdw_) of −60.31 ± 4.84 kcal/mol, an electrostatic interaction (ΔG_Coulomb_) of −35.83 ± 23.2 kcal/mol, a polar solvation free energy (ΔG_Solv_) of 51.66 ± 18.58 kcal/mol, a lipophilic free energy (ΔG_Lipo_) of −16.55 ± 1.39 kcal/mol, a hydrogen bonding free energy (ΔG_Hbond_) of −3.49 ± 1.45 kcal/mol, and a total free energy (ΔG_bind_) of −62.11 ± 9.03 kcal /mol. The total binding free energy was negative, indicating that the binding of the QEGPFVR–Keap1 protein complex was spontaneous. Together, these results suggested that QEGPFVR could bind spontaneously to Keap1 protein to form a complex, which was driven mainly by van der Waals forces and electrostatic interactions, and this binding was a non-covalent interaction.

To confirm the key residues involved in QEGPFVR interacting with Keap1 protein, the energy decomposition of the binding site of QEGPFVR with Keap1 protein was performed. The results are shown in Figure 9F. We only show the energy breakdown details of the 10 residues with the most potent binding energies for QEGPFVR interaction with Keap1 protein and the 4 amino acid residues with the highest contribution in the peptide QEGPFVR. QEGPFVR interacted with Tyr 334, Ser 363, Arg 380, Asn 382, Asn 387, and Arg 415 in the Keap1 protein active pocket, and Ser 555, Tyr 572, Phe 577, and Ser 602 amino acid residues, which are essential binding sites. The specific binding energies are shown in Table 3. Among them, the seventh amino acid residue Arg of QEGPFVR had the most potent van der Waals force (−8.59 kcal/mol) with Keap1 protein, the sixth amino acid residue Val had the most potent electrostatic attraction with Keap1 protein, and the highest total binding energy (−7.29 kcal/mol) was with Tyr 334. Van der Waals forces, hydrogen bonding, pi–pi stacking, electrostatic attraction, and lipophilic binding energy offset the unfavorable polar solvation energy contribution. Therefore, we hypothesized that amino acid residues Tyr 334, Ser 363, Arg 380, Asn 382, Asn 387, Arg 415, Ser 555, Tyr 572, Phe 577, and Ser 602 in the active pocket of Keap1 protein, as well as the amino acid residues Pro, Phe, Val, and Arg of the peptide QEGPFVR, synergistically promoted the binding stability of the QEGPFVR–Keap1 protein complex.

## 4. Discussion

Most peptides with high antioxidant bioactivity are oligopeptides with less than 10 amino acid residues, and peptides with a higher content of hydrophobic amino acids in the sequence have higher antioxidant activity [46]. In some low-molecular-weight antioxidant peptides, the presence of hydrophobic amino acids such as Leu, Iso, and Val can make the peptide chain more easily exposed to the solution and thus more effectively contact free radicals and scavenge them [47]. Moreover, hydrophobic amino acids help peptides to interact with lipid molecules to better inhibit lipid peroxidation reactions. By binding to lipid peroxides or scavenging the free radicals that trigger lipid peroxidation, the production of oxidation products is reduced, protecting cells and tissues from oxidative damage [48]. Peptides containing aromatic hydrophobic amino acids such as phenylalanine and tyrosine have an aromatic ring structure that can provide electrons or hydrogen atoms to free radicals, forming relatively stable free radical intermediates by themselves, thus terminating the chain reaction of free radicals and enhancing the antioxidant activity of peptides [49]. The presence of basic amino acids such as arginine in peptides can stabilize the secondary or tertiary structure of peptides through the formation of hydrogen bonds, salt bridges, and other interactions, which can help to maintain the conformation of the antioxidant active sites of peptides and enable them to bind and scavenge free radicals more effectively [50]. During the antioxidant process, the carboxyl groups on the side chains of acidic amino acids such as glutamic acid and aspartic acid can release protons to bind with free radicals, reducing the free radicals to more stable substances, thus exerting antioxidant effects [51]. This study showed that AQRDAGLL, QEGPFVR, and DVPPPRGPL all possessed lipid-lowering effects and antioxidant activities (Figure 4 and Figure 5). QEGPFVR, in particular, had the best activity among the three antioxidant peptides; however, this finding was inconsistent with that predicted by the evaluation system (Table 2). This may be because the active peptide DVPPPRGPL contains more hydrophobic amino acid residues, which, as a hydrophobic peptide, may only be partially soluble in an aqueous solution, resulting in the amount of active peptide able to contact and act on the cells being much lower than the theoretical added amount, thus affecting its effect on the cells. DVPPPRGPL can be chemically modified in later experiments to improve its solubility.

When cells are in an unfavorable environment, like in the presence of a large amount of ROS, cells initiate adaptive mechanisms to resist oxidative stress damage. As a source of oxidative stress, ROS can stimulate the dissociation of Nrf2 from Keap1 and prevent the ubiquitination degradation of Nrf2, which leads to the increase in Nrf2 expression [52]. After Nrf2 enters into the nucleus, its bZIP (basic leucine zipper) structural domain can bind to the ARE, which has a high degree of specificity. It can recognize and bind to the promoters of genes such as HO-1 and NQO1, thereby initiating transcription of these genes [53]. By increasing the expression of Nrf2, cells can enhance the transcription of antioxidant- and detoxification-related genes and synthesize more antioxidant enzymes and detoxification proteins, thereby mitigating cellular damage caused by harmful factors. This adaptive response helps to maintain cell survival and function to a certain extent [54].

MDS can simulate the interaction between small molecules and proteins [55]. Analyzing the energy changes and atomic trajectories during the reaction by observing the conformational changes of the active sites of proteins before and after binding to the ligands thus reveals the interaction mechanism between small molecules and proteins [56]. Some scholars used molecular dynamics simulation to screen chia seed peptides with anti-ACE activity [57]. The interaction between active peptides and proteins was clarified through MDS, and the rational design and modification of novel antioxidant peptides provided a key theoretical basis.

In the normal physiological state, Nrf2 is mainly bound to Keap1 and localized in the cytoplasm [58]. When the cell is in a normal state, Keap1 binds to Nrf2 through its multiple structural domains, restricts Nrf2 to the cytoplasm, and promotes ubiquitination degradation of Nrf2, thereby maintaining a low activity level of Nrf2 [59]. The ability of antioxidant peptides to bind to Keap1 is mainly based on structural complementarity and chemical interactions [40]. Antioxidant peptides have specific amino acid sequences and spatial conformations, and these structural features enable them to fit into specific binding sites on Keap1 [60]. For example, some antioxidant peptides may have regions rich in hydrophobic amino acids that can interact with hydrophobic pockets on the surface of Keap1 protein [61]. At the same time, the charged amino acids in the peptide chain may form electrostatic interactions with oppositely charged regions on Keap1 protein. In addition to electrostatic interactions, hydrogen bonding is essential in binding antioxidant peptides to Keap1 [62]. Functional groups such as hydroxyl (-OH) or amino (-NH_2_) groups in antioxidant peptides can form hydrogen bonds with carbonyl (-C=O) or imino (-NH) groups such as those in Keap1 protein. The formation of these hydrogen bonds helps to stabilize the peptide–protein complex’s structure [63]. In this study, van der Waals energy and electrostatic interactions in the antioxidant peptide QEGPFVR–Keap1 protein complex offset the unfavorable polar solvation energy contribution and were the main drivers of binding (Figure 8).

Protein–ligand interactions are classified into four types: hydrogen bonding (H-bonding), hydrophobic interactions, ionic bonds, and water bridges [64]. H-bonding plays a vital role in ligand binding and strongly influences drug specificity, metabolism, and adsorption, making it essential to consider hydrogen bonding properties in drug design [65]. Hydrophobic interactions typically involve hydrophobic amino acid and aromatic amino acid residues, or aliphatic groups on the ligand, and by extension, this category also includes cationic interactions [66]. Ionic or polar interactions are interactions between two oppositely charged atoms that do not involve hydrogen bonding [67]. Water bridges are hydrogen-bonded protein–ligand interactions mediated by water molecules, with slightly relaxed hydrogen bonding geometries compared to the standard H-bond definition. The present results show that hydrogen bonding, hydrophobic interactions, and water bridges mainly maintain the interaction between Keap1 and QEGPFVR. Therefore, QEGPFVR has the potential to regulate the function of Keap1 protein, which is likely to be a key target sequence to intervene in the physiological process of related cells (Figure 9). It is worthwhile to explore the value of QEGPFVR in the corresponding signaling pathway in the future.

However, there are limitations in utilizing peptides in molecular docking studies with Keap1 proteins. These include that most molecular docking studies are conducted in vitro under relatively simple experimental conditions, which cannot fully simulate the complex physiological environment of the cell, leading to the deviation of the docking results from the actual situation; molecular docking experiments often make it difficult to directly access the dynamic information of biological processes; and the analysis of molecular docking data usually fails to directly reveal complex biological network relationships. The limitations of Keap1–polypeptide docking are essentially a combination of the complexity of its target biology and technical bottlenecks in peptide drug development. In the future, with the development of precise design tools and efficient delivery platforms, Keap1-targeted peptides are expected to realize translational breakthroughs in the field of antioxidant therapy. For example, designing peptides targeting both the Kelch and BTB structural domains of Keap1 will enhance Nrf2 release through a metastable effect. Enzyme-responsive peptide precursors may be designed for lesion site-specific release. Coupling Keap1-targeting peptides with antioxidant enzymes may synergistically reduce ROS levels through multiple pathways. In addition, in vitro models (HepG2 cells), while simplifying mechanistic studies, have shortcomings, including the inability to simulate endocrine signaling from the liver to fat and the gut and the inability to assess systemic side effects. Animal models are therefore needed to validate the role of the antioxidant peptide QEGPFVR in NAFLD.

## 5. Conclusions

**Figure 10:**
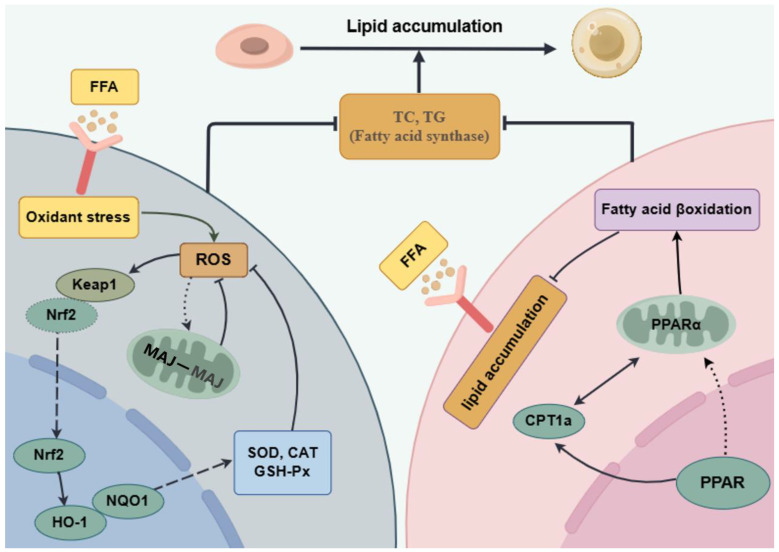
Overview of the roles of Nrf2, HO-1, NQO1, PPAR-α, CPT-1a, and MCJ in lipid accumulation and oxidative stress in HepG2 cells.

In this study, three peptides were identified from IV2 by reversed-phase LC-MS/MS: AQRDAGLL, QEGPFVR, and DVPPPRGPL. We systematically investigated the mechanisms of antioxidant and anti-lipid accumulation effects of the *Litsea cubeba* antioxidant peptides on a free fatty acid-induced HepG2 cell model. The results indicated that AQRDAGLL, QEGPFVR, and DVPPPRGPL enhanced HO-1 and NQO1 by regulating the dissociation of Nrf2 from Keap1, thereby ameliorating oxidative stress damage and lipid accumulation levels in FFA-induced HepG2 cells. The JC-1 fluorescence results showed that after AQRDAGLL, QEGPFVR, and DVPPPRGPL treatment, the HepG2 cell mitochondrial membrane potential increased, indicating that mitochondrial dysfunction was alleviated (Figure 10). In addition, AQRDAGLL, QEGPFVR, and DVPPPRGPL activated PPAR-α and CPT-1a, promoting fatty acid β-oxidation and lipid metabolism. MD verified the mechanism of action of QEGPFVR in alleviating oxidative stress. Molecular dynamics simulations showed that QEGPFVR could form a stable complex with Keap1 protein. The main driving force for binding was van der Waals forces, followed by electrostatic interactions. This discovery opens up new ideas and directions for developing QEGPFVR as a novel antioxidant drug class and exploring dietary supplements.

## Figures and Tables

**Table 1:** The primer sequences.

Gene	Forward Sequence (5′–3′)	Reverse Sequence (3′–5′)
Nrf2	TGTCTTAATACCGAAAACAAGCAGC	GACCACAGTTGCCCACTTCTTTT
HO-1	GCTAAGACCGCCTTCCTGCT	ACGAAGTGACGCCATCTGTGA
NQO1	TATCACCACTGGGGGTAGCG	GGAGTGTGGCCAATGCTGTAA
MCJ	AAGTAATCACGGCAACAGGG	AATAAAAGCCTGGCAGCCTTGC
PPAR-α	TTTCACAAGTGCCTGTCTGTCG	TCTTCAGGTAGGCTTCGTGGAT
CPT-1a	GCCATGAAGCCCTCAAACAG	CACCCACCACCACGATAAGC
GAPDH	CCTCGTCCCGTAGACAAAATG	TGAGGTCAATGAAGGGGTCGT

**Table 2:** Prediction of peptide activity.

Sequence	Predicted Score	Percentage of Hydrophobic Amino Acids (%)
LSPRDAVGH	0.325905	44.44
NAQRDAGIV	0.176496	44.44
EQRPTYSN	0.131206	12.5
EGDVFAVPR	0.457817	55.56
NAQRDAGLL	0.467791	44.44
HQEGPFVR	0.472375	37.5
QEGPFVRV	0.452377	50
DAGIVEGPK	0.222096	44.44
ALVVDGK	0.129537	57.14
ESEREVQ	0.0394552	14.29
LAGRDNIY	0.211819	37.5
QEGPFVR	0.513535	42.68
NAQRDAGIVEGPK	0.185251	38.46
AQRDAGLL	0.581992	50
AQRDAGIVEGPKGS	0.167792	35.71
RDAGIVEGPKGS	0.142415	33.33
EEVVHPY	0.0867931	42.86
GFDERVL	0.36642	42.86
DVPPPRGPL	0.837006	66.67
AGIVEGPKG	0.298716	44.44

**Table 3:** Decomposition energies of residues in QEGPFVR to Keap1 protein (kcal/mol).

Residues	Gbind	Coulomb	Solv	vdW	H-bond	Lipo	Pi–Pi
PeptideAA_4 (Pro)	−4.16	−1.2	−0.6	−4.5	−0.35	−0.63	0
PeptideAA_5 (Phe)	−5.97	−5.12	7.08	−6.69	−0.15	−3.4	−1.13
PeptideAA_6 (Val)	−4.98	−7.54	8.71	−6.27	−0.38	−1.41	0
PeptideAA_7 (Arg)	−4.73	0.8	3.04	−8.59	−0.74	−2.95	−0.45
Keap1_334 (Tyr)	−7.29	−1.94	2.26	−3.43	−0.08	−3.19	−0.91
Keap1_363 (Ser)	−2.53	−1.35	0.05	−1.06	0	−0.18	0
Keap1_380 (Arg)	−4.62	−12.7	11.58	−2.71	−0.27	−0.39	−0.14
Keap1_382 (Asn)	−5.42	−2.76	1.44	−2.95	−0.36	−0.47	−0.31
Keap1_387 (Asn)	−1.73	−1.7	1.82	−1.51	−0.14	−0.16	−0.05
Keap1_415 (Arg)	−2.26	−3.62	4.3	−2.1	−0.02	−0.82	−0.01
Keap1_555 (Ser)	−2.65	−2.42	0.8	−0.57	−0.4	−0.05	0
Keap1_572 (Tyr)	−4.94	−1.23	1	−2.66	−0.07	−1.59	−0.4
Keap1_577 (Phe)	−1.87	−0.24	0.18	−1.04	0	−0.77	0
Keap1_602 (Ser)	−5.32	−4.88	1.47	−1.39	−0.25	−0.28	0

## Data Availability

The original manuscript of this study is included in the article and further information is available upon reasonable request to the corresponding authors.

## Abbreviations

CAT: catalase; CPT-1a, carnitine palmitoyl transferase 1a; Da, Dalton; FFA, free fatty acid; GSH, glutathione; HO-1, heme oxygenases-1; high-dose: prescribed dose 200 µmol/L; IV2, *Litsea cubeba* multidimensional peptide; Keap1, kelch-1ike ECH-associated protein l; JC-1, mitochondrial membrane potential assay; low-dose: prescribed dose 50 µmol/L; MCJ, methylation regulates J protein; MD, molecular docking; MDA, lipid peroxidation; MDS, molecular dynamics simulation; NQO1, recombinant NADH dehydrogenase, quinone 1; Nrf2, nuclear factor erythroid 2 like 2; PPAR-α, peroxisome proliferator-activated receptor α; PPc, polyene phosphatidylcholine capsules; ROS, reactive oxygen species; SOD, superoxide dismutase; TC, total cholesterol; TG, triglyceride.
